# *Schistosoma* antigens: A future clinical magic bullet for autoimmune diseases?[Fn FN1]

**DOI:** 10.1051/parasite/2024067

**Published:** 2024-10-31

**Authors:** Mphatso Mayuni Chaponda, Ho Yin Pekkle Lam

**Affiliations:** 1 Master Program in Biomedical Sciences, School of Medicine, Tzu Chi University Hualien Taiwan; 2 Department of Biochemistry, School of Medicine, Tzu Chi University Hualien Taiwan; 3 Institute of Medical Science, Tzu Chi University Hualien Taiwan

**Keywords:** Immunotherapy, Autoimmune diseases, Schistosomiasis, Soluble egg antigen, Schistosome

## Abstract

Autoimmune diseases are characterized by dysregulated immunity against self-antigens. Current treatment of autoimmune diseases largely relies on suppressing host immunity to prevent excessive inflammation. Other immunotherapy options, such as cytokine or cell-targeted therapies, have also been used. However, most patients do not benefit from these therapies as recurrence of the disease usually occurs. Therefore, more effort is needed to find alternative immune therapeutics. *Schistosoma* infection has been a significant public health problem in most developing countries. *Schistosoma* parasites produce eggs that continuously secrete soluble egg antigen (SEA), which is a known modulator of host immune responses by enhancing Th2 immunity and alleviating outcomes of Th1 and Th17 responses. Recently, SEA has shown promise in treating autoimmune disorders due to their substantial immune-regulatory effects. Despite this interest, how these antigens modulate human immunity demonstrates only limited pieces of evidence, and whether there is potential for *Schistosoma* antigens in other diseases in the future remains an unsolved question. This review discusses how SEA modulates human immune responses and its potential for development as a novel immunotherapeutic for autoimmune diseases. We also discuss the immune modulatory effects of other non-SEA schistosome antigens at different stages of the parasite’s life cycle.

## Abstract (fr)

Les maladies auto-immunes sont caractérisées par une immunité dysrégulée contre les auto-antigènes. Le traitement actuel des maladies auto-immunes repose en grande partie sur la suppression de l’immunité de l’hôte pour prévenir une inflammation excessive. D’autres options d’immunothérapie, telles que les thérapies à base de cytokines ou à cellules ciblées, ont également été utilisées. Cependant, la plupart des patients ne bénéficient pas de ces thérapies car la maladie récidive généralement. Par conséquent, des efforts supplémentaires doivent être faits pour trouver des thérapies immunitaires alternatives. L’infection à *Schistosoma* est un problème de santé publique important dans la plupart des pays en développement. Les parasites *Schistosoma* produisent des œufs qui sécrètent en continu des antigènes solubles d’œufs (ASO), qui sont connus comme des modulateurs des réponses immunitaires de l’hôte en renforçant l’immunité Th2 et en atténuant les résultats des réponses Th1 et Th17. Récemment, les ASO se sont révélés prometteurs dans le traitement des troubles auto-immuns en raison de leurs effets immuno-régulateurs substantiels. Malgré cet intérêt, la façon dont ces antigènes modulent l’immunité humaine ne montre que des éléments de preuve limités, et la question de savoir si les antigènes de *Schistosoma* pourraient être utiles dans d’autres maladies à l’avenir reste sans réponse. Cette revue examine la manière dont les ASO modulent les réponses immunitaires humaines et leur potentiel pour le développement de nouveaux traitements immunothérapeutiques contre les maladies auto-immunes. Nous discutons également des effets immunomodulateurs d’autres antigènes de schistosomes non-ASO à différents stades du cycle de vie du parasite.

## Introduction

Although autoimmune diseases may be perceived as rare, it is estimated that one in ten people suffer from this condition [22], with significant mortality and morbidity. Autoimmune diseases can occur at any age, and in any gender or race [100], and they can range from organ-specific conditions like diabetes mellitus (DM), in which antibodies and T cells react to self-antigens in a specific tissue, to circulatory system disorders like systemic rheumatoid arthritis, in which antibodies react against antigens throughout the body [18]. Several factors are responsible for autoimmune diseases. The major attributing factors include genetic polymorphisms, such as human leukocyte antigens (HLA) alleles, and environmental factors, which include infections or ultraviolet (UV) irradiation. Nonetheless, dysregulated immune modulation is the primary reason for autoimmune diseases [18, 100]. Various immune cells have been associated with the pathogenesis of autoimmunity, including dendritic cells (DCs), macrophages, T cells, and B cells [57, 100]. DCs are antigen-presenting cells capable of differentiating naïve T cells into helper T (Th) cells and CD4^+^CD25^+^ regulatory T (Treg) cells [129]. Th cells can be differentiated into different subsets, including Th1, Th2, Th17, Th22, Th9, and Treg. Th1 cells are involved in cell-mediated inflammation and delayed hypersensitivity reactions. These cells are often defined by their production of IL-2 and interferon-gamma (IFN-γ). While IL-2 is essential for Treg proliferation and lineage survival [19]. IFN-γ is a multifunctional pro-inflammatory cytokine whose functions include activation of macrophage differentiation, enhancement of toll-like receptor (TLR) expression on immune cells, and antigen presentation [113]. IFN-γ has also been suggested to be associated with the pathology of autoimmune diseases [56]. Th2 cells are best known for producing IL-4, IL-5, and IL-13 and are associated with host defenses against parasites and involvement in allergies and atopic diseases such as asthma [136]. IL-4 is a cytokine with diverse functions essential for lymphocyte survival, plasma cell differentiation, and antibody class switch [62]. IL-4 has also been shown to promote the differentiation of macrophages and T-cell cells [62]. IL-4 has been suggested to be highly expressed in autoimmune diseases and, therefore, manipulating the effects of IL-4 offers good outcomes for immune-driven diseases such as allergy and cancer [62].

IL-5 and IL-13 have been found to play critical roles in the inflammation cascade by inducing B-cell class switching and IgE antibody production [76]. Both cytokines lead to an influx of eosinophils into the tissue, driving the pathogenesis of asthma and other airway inflammatory diseases [76]. While Th1 and Th2 immune cells counteract each other, an imbalance of Th1/Th2 has been found to be the cause of many autoimmune disorders.

Immunotherapies such as cytokine-targeted therapies, cell-targeted therapies, kinase-targeted therapies, and chimeric antigen receptor (CAR)-T cell therapy have long been used to fight against autoimmune diseases. For instance, the inhibition of IL-6 receptor and Janus kinase (JAK) is effective in treating patients with anemia and rheumatoid arthritis [89]; antibodies against IL-17 or IL-17 receptor have been used for psoriasis treatment [84]; CD19 CAR-T cell therapy, in addition to its application in B-cell lymphoma, has recently been used for patients with refractory systemic lupus erythematosus (SLE) [85]; low-dose IL-2 therapy also resulted in improvement in patients with SLE or psoriatic arthritis [60]. However, the proportion of patients achieving long-lasting remission by the current management is still low [130]. Therefore, more research is needed to find alternative immune therapeutics.

Schistosomiasis remains a major global health problem, affecting more than 200 million people worldwide [117]. In *Schistosoma* infection, the adult worms continuously lay eggs, which become trapped in organ tissues. Once trapped in the organ, schistosome eggs continuously release soluble egg antigen (SEA) that alters host immune response, leading to schistosomiasis. However, because of its immune-regulatory properties, SEA has been purified and used to treat various immune diseases [87]. In addition, other *Schistosoma* antigens such as Sm29, a native protein of the adult worm, also prompt a regulatory immune response that protects against exaggerated inflammatory responses [71]. In the current review, we attempt to provide an update on the immunomodulatory effect of SEA and other schistosome-related antigens, as well as their potential as an immunotherapeutic approach for different diseases.

## Immunopathology of schistosomiasis

Schistosomiasis is a very important tropical disease. It affects more than 200 million people worldwide and causes more than 300,000 deaths annually [117]. The disease is caused mainly by five species of schistosome, including *Schistosoma mansoni* (*S. mansoni*), *Schistosoma haematobium* (*S. haematobium*), *Schistosoma japonicum* (*S. japonicum*), *Schistosoma intercalatum* (*S. intercalatum*), and *Schistosoma mekongi* (*S. mekongi*) [80]. Once the cercariae penetrate human skin, they migrate into the bloodstream, becoming schistosomula, where further migration occurs at venous circulation to the liver portal vein, where they mature into adult worms. Male and female adult worms reside and copulate in the mesenteric venules. *Schistosoma mansoni* and *S. japonicum* are more frequently found in the inferior and superior mesenteric veins of the intestine, whereas *S. haematobium* most often inhabits the vesicular and pelvic venous plexus of the bladder. The paired adults then lay eggs that, besides being shed in stools (*S. mansoni* or *S. japonicum*) or urine (*S. haematobium*), enter the circulation and become trapped in the liver, intestine, or other organs. The entrapment of eggs in organ tissues leads to granulomatous inflammation and subsequently fibrosis.

It is acknowledged that schistosomiasis is not caused by the worms themselves but by the body’s reaction to the eggs. Trapped eggs continuously release soluble egg antigen (SEA). The body’s initial immune response against SEA involves local secretion of Th1 cytokines such as TNF-α and IL-2 [72], leading to monocyte, neutrophil, and lymphocyte infiltration. The influx of these cells results in phagocytosis and granuloma formation. Omega-1, a glycosylated T2 ribonuclease (RNase) from one of the many components of SEA, activates dendritic cells (DCs), which promotes a shift towards a Th2 immune response [37]. This shift from a Th1- to Th2-skewed response significantly contributes to liver fibrosis in schistosomiasis, characterized by a decrease in IFN-γ (Th1 cytokine) and an increase in IL-4, IL-5, and IL-13 (Th2 cytokines) profiles [134]. IFN-γ suppresses hepatic stellate cell (HSC) activation [11], while IL-4 and IL-13 induce its activation [43]. Together, the imbalance of these cytokines gives rise to the progression of liver fibrosis. Injection of mice with schistosome eggs and IL-12 (a Th1-inducing cytokine) has been shown to inhibit the Th1 to Th2 shift and ameliorates granuloma formation and fibrosis, suggesting that a Th2 response may be fundamental for schistosome-induced fibrogenesis [126]. It has been demonstrated that the drug praziquantel is effective in treating schistosomiasis by modulating cytokine responses [109]. Praziquantel increases serum levels of IFN-γ and inhibits IL-4 [109]. Although the increase of IFN-γ improves schistosomiasis, several studies have associated its upregulation with several human autoimmune diseases [45]. Furthermore, studies have shown that polarization towards either Th1 or Th2 extreme can also contribute to the pathogenesis of schistosomiasis. Therefore, striking a balance between a Th1/Th2 immune response is key in improving the clinical manifestations of schistosomiasis.

## *Schistosoma* soluble egg antigen (SEA) as an immunotherapy for autoimmune diseases

The immunoregulatory effects of soluble egg antigen (SEA) have led researchers to explore its potential therapeutic use in treating autoimmune and inflammatory disorders such as diabetes, colitis, and multiple sclerosis [20]. A summary of how SEA influences host immunity and autoimmune diseases is provided in Table 1.

**Table 1:** A summary of schistosome products with immunomodulatory effect against autoimmune diseases.

Schistosome product		Diseases	Immunomodulatory effect	References
Egg	Soluble egg antigen (SEA)	Graves hyperthyroidism	Suppresses Th1-type anti-TSHR IgG2a autoantibodies and IFN-γ secretion	[88]
		Asthma	Downregulates Th2 response and upregulates Th1 response, increases IFN-γ production	[97]
		Type 1 and Type 2 Diabetes	Induces Th2 response	[115]
			Stimulates IL-33 secretion and increases Tregs	[48]
		Inflammatory bowel diseases	Increases FoxP3^+^ Treg cells and secrets Th2 cytokines	[49]
		Skin transplantation	Increases CD4^+^IL-4^+^ T cells and CD4^+^Foxp3^+^ T cells but decreases CD4^+^IFN-γ^+^ T cells in the skin transplant	[59]
		Autoimmune encephalomyelitis	Induces Th2-dominant response and reduces leukocyte infiltration in the CNS	[20]
	Interleukin-4 inducing principle from Schistosoma mansoni eggs (IPSE/alpha-1)	Allergic airway inflammation	Induces Bregs which activates Tregs	[65]
	28-kilodalton glutathione S-transferases (28GST)	Schistosomiasis	Induces Th1 response	[67]
		Colitis	Downregulates Th1 and Th17 and activates M2 macrophages and Th2 responses	[106]
	*Schistosoma japonicum* HSP60-derived peptide (SJMHE)	Delayed-type hypersensitivity	Induces CD4^+^CD25^+^ Tregs with overexpression of CTLA-4, IL-10, and TGF-β1	[121]
		Inflammatory bowel diseases	Increases Th2 and Treg cells, upregulates IL-10 and reduces Th17 and IL-17	[108]
		Allergic rhinitis	Upregulates Bregs with IL-10 production	[42]
	Schistosoma mansoni 14-kDa fatty acid-binding protein (Sm14)	Schistosomiasis	Activates CD4^+^ T lymphocytes and produces higher IFN-γ and TNF-α	[8]
			Increases production of IgG specific antibodies, IL-2, TNF-α, and IFN-γ	[104]
	Omega-1 (ω1)	Diabetes	Regulates an inflammasome-dependent IL-1β and triggers Tregs production	[55]
		Human immunodeficiency virus infection	Induces CD4^+^ T cells and stimulates Th2 responses	[86]
	*Schistosoma mansoni* major egg antigen (Sm-p40)	Hypertension	Reduces Caveolin-1 expression by stimulating TLR-4/CD14-mediated phosphorylation	[75]
Cercariae	Cercarial antigen	Arthritis	Increases serum levels of IL-10 and IFN-γ; increases Tregs and reduces IL-17	[35]
		Colitis	Induces macrophage-dependent response	[112]
	KS-84 (a synthetic peptide of Sm16)	Liver fibrosis	Downregulates TGF-β expression	[16]
	*Schistosoma mansoni* Cathepsin B 1 (SmCB1)	Schistosomiasis	Increases production of IL-5 and IL-13 and decreases IFN-γ secretion	[114]
Adult worm	*Schistosoma mansoni* Kunitz type serine protease inhibitor (SmKI-1)	Acetaminophen-induced liver injury	Reduces neutrophil recruitment and elastase activity	[83]
		Pleural cavity inflammation	Lowers leukocyte infiltration	[83]
	*Schistosoma mansoni* protein 29 (Sm29)	Leishmaniasis	Increases CD4^+^CD25 and CD4^+^CTLA-4^+^ T cells and increases IL-10	[71]
		Allergic airway inflammation	Decreases IgE levels and increases the numbers of CD4^+^FoxP3^+^ T cells	[15]
		Human T cell lymphocytic virus type 1	Induces IL-10 and reduces IFN-γ	[69]
	*Schistosoma japonicum* tetraspanin orphan receptor (SjTOR)	Schistosomiasis	Modulates complement pathway; induces IgG1 and IgG2a antibodies	[73]

### Immune modulation by SEA

**Figure 1:**
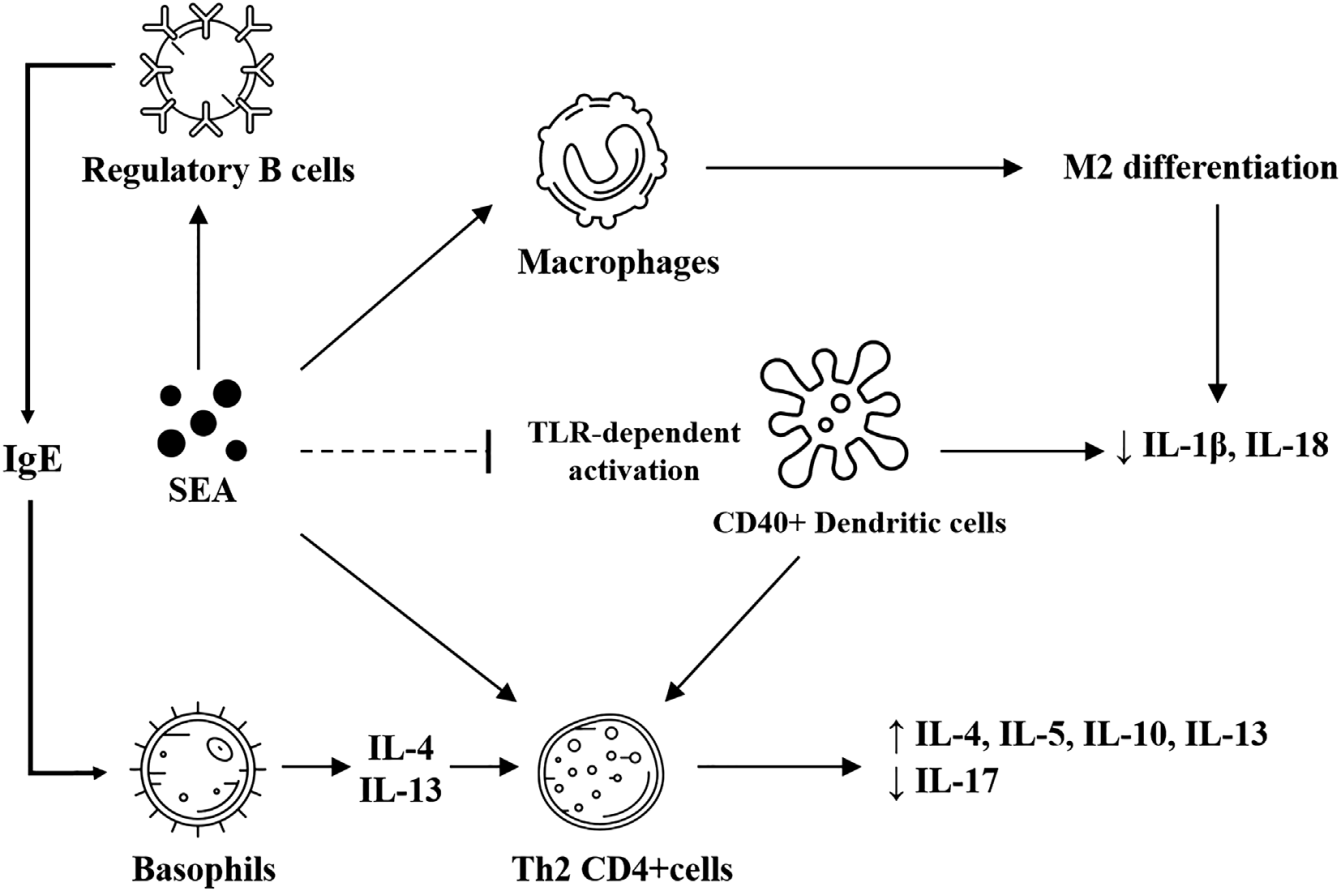
Immune responses induced by SEA. SEA induces M2 differentiation of macrophages and prevents toll-like receptor-dependent activation of dendritic cells, which is correlated with reduced production of inflammatory cytokines. Dendritic cells also induce the differentiation of CD4^+^ T cells into the Th2 subset through a CD40-dependent mechanism. SEA can also activate Th2 CD4^+^ T cells and regulatory B cells. Regulatory B cells, once activated, secrete IgE and further stimulate basophil production of IL-4 and IL-13, which then induce Th2 CD4^+^ T cells. Th2 CD4^+^ T cells can release various Th2 cytokines such as IL-4, IL-5, IL-10, and IL-13, shifting the overall immunity into a Th2-dominant response.

SEA exhibits a powerful immune-modulatory effect (Fig. 1); therefore, researchers have begun investigating its immune mechanism. In mice, injection with SEA has been shown to induce a higher number of Th2 cells, producing higher levels of IL-4, IL-5, and IL-13. SEA treatment also decreases IL-17 secretion from CD4^+^γδ^+^ T cells [74]. This is positively linked to tissue repair of murine muscle injury, and negatively linked to fibrogenesis in disease models of the corneal and articular joints [74]. SEA can induce M2 differentiation of macrophages via signal transducer and activator of transcription (STAT)-6 and phosphatidylinositol 3-kinase (PI3K)-dependent pathways [116]. SEA treatment also prevents toll-like receptor (TLR)-dependent activation of DCs, as confirmed by the lack of major histocompatibility complex (MHC) upregulation, CD80/CD86 upregulation, and Th1 and Th17 cytokine production [20]. When SEA-treated DCs were injected into mice, they drove the differentiation of naïve T-cells into Th2 cells and produced higher IL-4, IL-5, and IL-10 [20]. Notably, the induction of tolerogenic DCs by SEA depends on CD40, as the absence of CD40 fails to develop Th2 responses in mice induced by SEA-exposed DCs [20]. On a molecular level, the interaction between glycosylated SEA and DCs results in increased expression of suppressor of cytokine signaling1 (SOCS1) and SH2-containing protein tyrosine phosphatase-1 (SHP1), two proteins that inhibit TLR4 signaling [64]. Although it is still unclear how SEA-induced DCs drive Th2 differentiation, recent literature has suggested that CD40, CD252, and nuclear factor κB (NFκB) are required in the process [20]. SEA has also been found to inhibit inflammatory reactions by interacting with the B-cell lymphoma-3 (BCL-3) protein in DCs [64]. In addition to DCs, studies have suggested that SEA can internalize into regulatory B (Breg) cells and induce significant production of IL-10 and immunoglobulin E (IgE) [20]. At the same time, the Th2 response has long been considered an anti-schistosome response, although this response consequently leads to granuloma formation and fibrogenesis in natural infection (where the eggs persist in the tissue). It is now hypothesized that schistosome antigens or their derived products may induce this immune signature to repair autoimmunity-associated damage or reverse the pathogenesis of some diseases.

### Graves hyperthyroidism

Graves hyperthyroidism is an autoimmune disease that affects the thyroid gland. The disease is characterized by lymphocytic (mainly T lymphocytes) infiltration of thyroid parenchyma [5]. A Th1 immune response promotes the production of IFN-γ and TNF-α, which activates thyrocytes to secrete CXCL10, aggravating the disease [5]. Further, this leads to autoantibody production against the thyroid-stimulating hormone receptor (TSHR), resulting in the overproduction of thyroid hormones [26]. The consequence is symptoms such as goiter, irregular heartbeat, and ophthalmopathy [26]. An earlier report indicated that SEA suppresses the production of Th1-type anti-TSHR IgG2a autoantibodies and IFN-γ during Graves hyperthyroidism, which decreases the severity of the disease [88]. However, it has also been shown that despite the induction of anti-TSHR immune response in mice, SEA was ineffective in curing the disease [88]. The full extent of the anti-TSHR immune reactions induced by SEA on Graves hyperthyroidism is unclear, as it has not been studied extensively. Further research on the immune effects of SEA on Graves hyperthyroidism could provide valuable insight that could aid in developing new immunotherapies for this common autoimmune disease.

### Asthma

Asthma is a prevalent but non-communicable disease affecting 300 million individuals worldwide [30]. It poses a significant economic burden and has high mortality rates. Although asthma is not classified as an autoimmune disease, it does entail a dysregulation of the immune system. The immune signature of asthma involves eosinophilia, IgE induction of airway smooth muscle, and increased levels of IL-4, IL-5, and IL-13 [78]. Therefore, targeting the Th2 responses could be an effective way to combat asthma. Although SEA has been shown to induce Th2 response, new T-cell epitopes identified on *S. japonicum* protein 40 (Sjp40), one of the components of SEA, have been shown to enhance Th1 response by increasing IFN-γ and suppressing Th2 responses, thereby alleviating allergic asthma in a mouse model [97]. Interleukin-4 inducing principle from *S. mansoni* eggs (IPSE/alpha-1), a glycoprotein of SEA, increases IL-10 production from Bregs in mice and humans, reducing experimental allergic airway inflammation [46].

Interestingly, multiple comparative studies investigating the relationship between asthma and *S. mansoni* infection in endemic settings suggested a significant inverse correlation between asthma and infection by *S. mansoni* [95]. It has been demonstrated that *S. mansoni* may suppress immediate hypersensitivity reactions, leading to a less severe form of asthma.

### Type 1 and type 2 diabetes

According to the International Diabetes Federation (IDF), 451 million people were living with diabetes as of 2017 globally. Unfortunately, this number is expected to rise to 693 million by 2045 [4]. Chronic pancreatic islet inflammation is a defining characteristic of both type 1 and type 2 diabetes. Research has demonstrated that IL-1β is responsible for type 1 and type 2 diabetes by overstimulating the β-cells of the pancreatic islets of Langerhans [32]. Furthermore, it has been described that IL-1β induces the production of other cytokines and chemokines, including IL-6, IL-8, IL-33, TNF, and CC-chemokine ligand 2 (CCL2) [32]. These mediators attract various immune cells into the islets, leading to chronic inflammation and a harmful cycle of auto-stimulation of IL-1β [32]. Imbalances in Th1, Th17, and Tregs cells have been described to lead to the pathogenesis of diabetes [7, 138]. Thus, maintaining a balance between these T cells is essential for controlling both type 1 and type 2 diabetes.

Single cytokine blockage has shown limited effectiveness as a standalone treatment for diabetes [32], indicating a need for further research into alternative immune therapies. It was shown that *S. mansoni* infection in obese mice led to body weight reduction, lower insulin resistance, and lower glucose intolerance [54]. *Schistosoma japonicum* soluble egg antigen was previously found to increase Th2 immune response and Tregs, leading to improved type 2 diabetes in Lepr^db/db^ mice [115].

SEA-derived omega (ω)-1 protein has also been suggested to improve the metabolic status of obese mice by binding to CD206 and stimulating the release of IL-33, a Th2 cytokine inducer [48]. Although the induction of Tregs and Th2 cytokine responses have been shown to improve diabetes, there is no direct evidence on whether they are associated with IL-1β.

### Inflammatory bowel diseases

Inflammatory bowel disease (IBD) is a chronic inflammatory state of the gastrointestinal tract and is classified into two main clinical conditions: ulcerative colitis and Crohn’s disease. IBD is characterized by chronic inflammation and a dysregulated inflammatory immune response. T helper cells play a crucial role in the pathogenesis of IBD as they are known to differentiate based on their surrounding environment [44]. Multiple studies have demonstrated that IL-17, a pro-inflammatory cytokine secreted by Th17 cells, is the primary driver of IBD [44]. The differentiation of Th17 and Treg cells is related, as these cells share a common signaling pathway mediated by TGF-β [137]. The induction of Tregs under the influence of IL-10 improves clinical symptoms of IBD in an animal model [133]; therefore, maintaining the Th17/Treg cell balance is crucial in preventing IBD [128].

Previously, SEA has been shown to significantly reduce the severity of 2,4,6-trinitrobenzene sulfonic acid (TNBS)-induced colitis [51] and dextran sulfate sodium (DSS)-induced colitis [49] in a mouse model by increasing the number of FoxP3^+^ Treg cells and secretion of Th2 cytokines, including IL-10 [49].

### Organ transplantation

Although *Schistosoma*-infected patients are not included in transplant donor acquisition, some studies have suggested that organ transplantation from donors with schistosomiasis has been successfully performed without any short-term or long-term adverse effects [23]. Additionally, evidence indicates that *Schistosoma* infection does not pose a significant risk for transplantation, as infected organ recipients do not appear to experience any harmful consequences [68]. Studies have shown that graft rejection comprises an infiltration of various inflammatory cells such as monocytes, DCs, NK cells, eosinophils, and CD8^+^T cells [41]. It has been found that a proper balance of Tregs and Th17 cells is critical for graft tolerance, as Th17 cells contribute to chronic graft rejection, whereas Tregs promote immune suppression and graft tolerance [52]. SEA has been demonstrated to suppress skin graft rejection and prolonged survival by regulating IFN-γ and limiting the inflammatory effect of Th1 and Th17 cells [59]. SEA treatment also led to higher CD4^+^IL-4^+^ T cells and CD4^+^Foxp3^+^ T cells and decreased CD4^+^IFN-γ^+^ T cells within the skin transplant [59]. These findings suggest that SEA, through its immune-regulatory effect, could be a viable treatment in preventing organ transplant rejection.

### Multiple sclerosis

Multiple sclerosis (MS) is characterized by the infiltration of autoreactive immune cells into the central nervous system (CNS), causing neuronal damage [29]. Several Th cells, such as Th1, Th17, and Th22, have been associated with MS [118]. Tregs inhibit the infiltration of effector T cells into the CNS in mice with experimental autoimmune encephalomyelitis (EAE) [118]. Additionally, Tregs have been shown to inactivate mast cells, which are reported to worsen the symptoms of MS [118].

While no studies have directly examined the effects of SEA in MS, studies have been conducted on the potential impacts of schistosome eggs and cercariae in this disease. To this end, mice with EAE were treated with schistosome eggs before disease induction. The results lead to significant protection from the disease, a shift from Th1-dominant response to Th2-dominant response, and reduced leukocyte infiltration in the CNS [20]. It was also shown that *S. mansoni*-infected EAE mice have a reduced Th1 response and CNS inflammation [20], providing a fundamental basis for future use of SEA or other schistosome-related products in MS.

### Cancers

As host immunity plays a significant role in regulating tumor cell growth and progression [50], there has been great interest in immunotherapies in treating cancers. Although immunotherapies for treating cancers and autoimmune diseases seek opposite effects on the immune system (one to enhance anti-tumor immunity and the other to reduce immune activation and suppress inflammation), they may involve the same immune pathways [1]. For example, interferon regulatory factor 4 (IRF4), a member of the IRF family of transcription factors, plays a crucial role in immune cell differentiation and function, including B-cells, T-cells, and DCs [127]. IRF4 has been found to facilitate the infiltration of CD8^+^ T cells, advancing both tumors and autoimmune diseases [127]. Therefore, eliminating IRF4 has been shown to impede tumor growth, enhance treatment for autoimmune disease, and promote organ graft tolerance [127]. This suggests that immunotherapies designed for autoimmune diseases could also be adapted to cancer treatment.

However, there are still challenges in applying immunotherapy to cancers. Certain non-immunogenic cancers, such as pancreatic cancer, hormone receptor-positive breast cancers, and glioblastoma, have been incredibly resistant to this approach. Failure of immunotherapy may even occur in immunogenic cancers, such as non-small cell lung cancer (NSCLC) [6] and multiple myeloma [98].

Currently, no research has yet investigated the effectiveness of SEA in preventing or treating cancer. However, SEA may serve as a therapeutic approach in cancer, especially when cercarial antigens have already been suggested to be able to treat colon cancers [34]. Despite SEA holding potential as a cancer treatment, further research is needed to comprehend its mechanisms and effects. *Schistosoma* infection and the secretions of *S. haematobium*-related egg antigens, such as IPSE/alpha-1, have been shown to stimulate continuous inflammatory responses that lead to bladder carcinogenesis [79, 105]. It has been suggested that the chronic inflammatory response caused by *S. japonicum* and *S. mansoni* infection provides a suitable environment for the occurrence of genomic instability, triggering the development of colorectal cancer [102].

On the contrary, it has also been shown that treating mice with *S. mansoni* antigens reduced the tumor number and tumor size of 1,2-dimethylhydrazine-induced colorectal cancer [34]. The link between schistosomiasis and cancer is not a simple cause-and-effect relationship because many other factors are also involved. For example, the co-occurrence of schistosomiasis and hepatitis B virus (HBV) infection plays a role in the development of hepatocellular carcinoma [63]. Currently, there is no sufficient evidence to associate other schistosome species apart from *S. haematobium* with cancer. Therefore, by using specific antigens from the egg, cancer therapy can be precisely timed and controlled for better outcomes.

## Immune modulatory effects of specific components within SEA

Although the term SEA is used as an acronym for soluble egg antigen, it is in fact not just a single antigen. SEA is a crude extract of schistosome eggs, involving disruption of the egg with a homogenizer using an extracting buffer (such as phosphate-buffered saline or other lysis buffers). The homogenate comprises complex components, not only proteins but also glycoproteins, polysaccharides, and glycolipids [24]. The components of SEA are therefore derived from the eggshell, the miracidium within the egg, and egg-secretory proteins [77].

While SEA has been found to possess immune regulatory properties in different diseases, it has also been shown to activate the NLRP3 inflammasome in hepatic stellate cells (HSCs) [81]. This activation enhances the secretion of IL-1β in the liver which may be an early mechanism to turn on inflammatory responses that lead to fibrosis; not to mention that IL-1β is a primary driver of many inflammatory diseases [81].

Therefore, there is a need for further research on SEA as a therapeutic agent by dissecting and analyzing different SEA components. Some of the thoroughly researched egg secretory proteins include but are not limited to interleukin-4 inducing principle from *S. mansoni* eggs (IPSE/alpha-1),Please check and approve the page number added in reference [25].28-kilodalton glutathione S-transferases (28GST), *S. japonicum* HSP60-derived peptide, *S. mansoni* 14-kDa fatty acid-binding protein (Sm14), omega-1 (ω1), *S. mansoni* large subunit calpain (Sm-p80), *S. mansoni* protein 40 (Sm-p40) and micro-exon gene proteins (MEGs). Proteomics analysis has provided us insights into the biological characteristics of these egg antigens and has revealed potential vaccine candidates for schistosomiasis [17]. For example, impartial phage display screening has identified Sm-p80 and MEG proteins as potential *S. mansoni* vaccine candidates in the rhesus macaques *Macaca mulatta* [123]. Therefore, further understanding of the immunoregulatory effects of each SEA component may enable us to clarify the therapeutic or pathogenic mechanism. Below, we will review the specific components of SEA and their immune modulatory effects. Table 1 describes the immune modulatory effect of these particular components of SEA on diseases.

### Interleukin-4 inducing principle from *Schistosoma mansoni* eggs (IPSE/alpha-1)

Interleukin-4 inducing principle from *S. mansoni* eggs (IPSE/alpha-1) is a major glycoprotein secreted by the eggshell of *S. mansoni* egg [61]. IPSE/alpha-1 is an immunoglobulin-binding protein that interacts with IgE, leading to basophil activation [61]. It has also been reported that the IPSE/alpha-1-activated basophils secret IL-4 and IL-13, two of the main drivers of Th2 response [65]. The production of IL-4 and IL-13 from basophils downregulates inflammatory responses in schistosomiasis as these cytokines result in the differentiation of monocytes to alternatively activated macrophages [65]. IPSE/alpha-1 stimulates IL-10 secretion from naïve B cells and induces differentiation of Breg cells [20]. Breg cells have been demonstrated to improve the outcome of autoimmune diseases such as EAE, collagen-induced arthritis, and autoimmune myocarditis (AEM) in animal models [124]. Breg cell induced by IPSE/alpha-1 also stimulates Treg cell development and alleviates experimental allergic airway inflammation [46]. However, IPSE/alpha-1 can potentially be associated with bladder cancer [79], and therefore, further research on this antigen is recommended.

### 28-kilodalton glutathione S-transferases (28GST)

28-kilodalton glutathione S-transferases (28GST) is an enzyme that neutralizes endogenous and exogenous free radicals and is present in all stages of the schistosome, except in intra-uterine immature egg [93]. In one study, 28GST was shown to induce a Th1 immune response, thereby protecting the host from *S. mansoni* infection [67]. However, in another study, 28GST downregulates Th1 and Th17 responses and induces activation of M2 macrophages and Th2 responses, thereby improving intestinal inflammation in mice with TNBS-induced colitis [106]. Similar results can be observed in colitic rats treated with 28GST, which improved their colitis symptoms by inducing Th2 immune responses and eosinophil infiltration [33]. Notably, 28GST used in these colitis studies was purified from *S. haematobium*. Although *S. haematobium* and *S. mansoni* differ in their parasitic location, they induced a very similar immune response in the host. Therefore, it is possible that antigens derived from *S. haematobium* or *S. mansoni* may show very similar immunomodulatory effects.

In a phase IIa clinical trial, the Anti-CROHN Enzymatic Molecule (ACROHNEM) program (ClinicalTrials.gov Identifier: NCT02281916), 28GST was applied in patients with Crohn’s disease. Among all ten patients recruited, eight received three subcutaneous injections of recombinant 28GST within three months, followed by a nine-month course investigation [13]. Injection of 28GST reduced disease activity scores in these patients by 30% with no adverse effects. Analysis of the patients’ fecal microbiota composition showed an increase in *Bifidobacterium*, a bacterium that exerts positive health benefits on its host, and a decrease in Veillonellaceae, bacteria associated with inflammatory events [40]. However, the major limitation of this study remains the small sample size, which may lead to possible biased interpretation; therefore, validation with a larger sample size could be done in the future. Nevertheless, these studies suggest that 28GST may be a beneficial therapeutic weapon for inflammatory bowel disease in the future.

### *Schistosoma japonicum* HSP60-derived peptide (SJMHE)

*Schistosoma japonicum* HSP60-derived peptide (SJMHE) is a peptide molecule of the heat shock protein family D (HSP60) member 1 (HSPD1) found in the SEA of *S. japonicum*. SJMHE1 inhibits delayed-type hypersensitivity in mice through induction of CD4^+^CD25^+^ Tregs with overexpression of CTLA-4, IL-10, and TGF-β1 [121]. By inhibiting the activity of Th1 and Th17 cells, SJMHE1 can lessen the severity of DSS-induced acute and chronic colitis in mice [108]. This improvement in IBD was accompanied by enhanced Th2 response, reduced IL-17 expression, and increased IL-10 expression [108]. Treatment of mice with SJMHE1 also resolved allergic rhinitis by upregulating IL-10-producing Breg cells [42].

### *Schistosoma mansoni* 14-kDa fatty acid-binding protein (Sm14)

Schistosomes do not process oxygen-dependent pathways to manufacture fatty acids and sterols; therefore, the parasite employs fatty acid-binding proteins, mainly by a 14-kDa polypeptide (Sm14), to internalize host fatty acids [3]. Sm14 is found in the egg and adult worm [9]. Immunization with recombinant Sm14 (rSm14) has been shown to protect against schistosomiasis in mice and rabbits, highlighting the potential of Sm14 as a vaccine candidate [3]. A study in Brazil, a country considered endemic for schistosomiasis, suggested that Sm14 protects uninfected individuals against schistosomiasis by stimulating CD4^+^ T cells and producing higher IFN-γ and TNF-α [8]. Previously, our study demonstrated that mice immunized with heat-killed *Cutibacterium acnes*-adjuvanted rSm14 increased humoral immune responses against *S. mansoni* and reduced *S. mansoni*-associated liver fibrosis in infected mice [66]. Our findings emphasize the critical role of using Sm14 with an adjuvant to induce a robust immune response against schistosomiasis. Additionally, a phase I clinical trial using Sm14 as a vaccine candidate in endemic areas has shown promising results in preventing schistosome infections. This clinical trial also showed that Sm14 elicited increased schistosome-specific IgG antibodies and a robust cytokine response of TNF-α, IFN-γ, and IL-2 in vaccinated individuals [104].

### Other schistosome-soluble egg antigens

Omega-1 (ω1) is a glycosylated secretory antigen of SEA. It is a hepatotoxic glycoprotein with ribonuclease (RNase) T2 activity. Omega-1 binds to the mannose receptor on DCs, thereby activating DCs [107]. The activation of DCs leads to the induction of Th2 immune responses. Both the RNase T2 activity and Th2 polarization contribute to granuloma formation [55]. It has been shown that knocking out the ω1 gene from schistosome eggs exhibited failure in Th2 polarization and reduced granuloma size [55]. Omega-1 also modulates the infection capacity of the human immunodeficiency virus (HIV) *in vitro* by stimulating DCs, inducing CD4^+^ T cells and stimulating Th2 response [86]. Omega-1 reduces the development of diabetes in NOD mice by regulating an inflammasome-dependent IL-1β release and triggering Tregs production [55].

*Schistosoma mansoni* large subunit calpain (Sm-p80) is a protein responsible for biogenesis and renewal of the surface membrane of schistosome [82]. The protein is present in all intra-mammalian parasite stages and is found on the surface of the parasite syncytium, making it a good candidate for schistosome vaccine. The protein is highly antigenic and is able to induce host immune responses. Several studies have reported that Sm-p80 vaccination protects against infection of different schistosomes species. Vaccination of Sm-p80 also improves hepatic, intestinal, and urogenital schistosomiasis [82].

*Schistosoma mansoni* major egg antigen (Sm-p40) is another major egg component of *S. mansoni*; it elicits a strong Th1 immune response in mice, suggesting its potential in treating autoimmune diseases [90]. *In vitro*, Sm-p40 stimulates TLR4/CD14-mediated transient phosphorylation of Caveolin-1 (Cav-1) at Tyr14 in human lung microvascular endothelial cells (HMVEC-L), leading to a reduction of Cav-1 expression [75]. Cav-1 has been implicated in the development of hypertension [120], suggesting the potential of Sm-p40 in treating hypertension.

Micro-exon gene proteins (MEGs) are a large family of *S. mansoni* proteins encoded by genes of various symmetrical micro-exons. MEG-2 and MEG-3 are upregulated and highly expressed in mature liver-entrapped eggs in *S. mansoni*-infected mice [91]. MEG-24 and MEG-27 are identified as α-helical membrane-active peptides located in the parasite sub-tegumental cells that could interact with the host immunity similar to other α-helical membrane-active peptides [38]. In addition, MEGs have gene expression patterns similar to omega-1 and IPSE/alpha-1, suggesting their possible mechanism in establishing immune modulation [91]. Using a microarray approach with more than 170,000 unique peptide sequences, MEG-12 was found to be a sensitive and specific immunogenic linear peptide that could be used as a diagnostic marker or to be included in a multi-epitope vaccine construct [119].

## Immune modulatory effect of other non-SEA *Schistosoma* antigens

In addition to egg-related antigens, studies also demonstrated the use of other *Schistosoma* antigens in treating autoimmune diseases. Here, we will discuss non-SEA *Schistosoma* antigens that can potentially regulate host immunity and their impact on diseases. Table 1 indicates the immune modulatory effects of the crude and specific antigens on different diseases.

### Immune modulation by *Schistosoma* cercarial antigens

Although the immune modulatory effect of SEA during schistosomiasis has been extensively researched, the immune regulatory effects of secretory cercarial antigens remain understudied. Current evidence supports the notion that infective cercariae employ several immunomodulatory mechanisms to penetrate host skin [58]. Cercariae alter host DCs, mast cells, and macrophages, leading to increased IL-10 production. Transformed cercarial antigens have been shown to activate bone marrow-derived DCs by increasing MHC-II, CD40, and CD86 expression and inducing IL-6 and IL-12p40 [58]. Lewis^X^ immune-modulating molecules isolated from schistosome eggs are also present in cercariae and have been reported to cause a suppressive immune response [58]. Heat-killed *S. mansoni* cercarial antigen improved arthritis in rats by maintaining the balance of pro-inflammatory and anti-inflammatory responses through increasing serum levels of IL-10 and IFN-γ, increasing Foxp3^+^ Tregs, and reducing IL-17 levels [35]. Mice infected with cercariae have also been shown to reduce DSS-induced colitis in a macrophage-dependent manner [112]. In the following, we will review specific cercarial antigens.

### *Schistosoma mansoni* protein antigen 16 (Sm16)

Sm16, a 16 kDa protein secreted by *S. mansoni* cercariae, is a major component of cercarial secretory antigens [25]. Sm16 modulates host skin immune response to aid successful cercarial penetration. Sm16 inhibits IFN-γ stimulation of monocytes *in vitro* in a TLR2-independent manner [103]. *In vitro*, recombinant Sm16 has been shown to induce pro-inflammatory macrophage response and decrease the production of lipopolysaccharide (LPS)-induced inflammatory cytokines [110]. Sm16 also increases the anti-inflammatory cytokine, interleukin-1 receptor antagonist (IL-1Ra), in keratinocytes [2]. Furthermore, Sm16 suppresses cutaneous inflammation by reducing neutrophil infiltration in mice [25].

KS-84, a synthetic peptide deriving from Sm16, has been shown to downregulate TGF-β1 gene expression in LX-2 cells, reducing liver fibrosis [16]. These studies suggest the immune modulatory effect of Sm16 and propose its potential use for immunotherapy.

### *Schistosoma mansoni* cercarial elastase (SmCE)

*Schistosoma mansoni* cercarial elastase (SmCE) is a proteolytic enzyme responsible for cercariae to degrade the skin barrier when invading host skin [47]. SmCE induces dermal DCs to express high levels of programmed death ligand (PD-L) and IL-10 to aid parasite invasion of host skin [122]. Therefore, immune responses targeting this enzyme are thought to protect against cercariae penetration [47]. Although SmCE is poorly immunogenic, multiple immunizations with this enzyme still protected the mice from *S. mansoni* infection. Furthermore, it has been found that immunizing the mice with recombinant SmCE (rSmCE), an inactive enzyme, induces specific IgG1 responses [36]. Mice vaccinated with rSmCE had lower worm and egg burden. Therefore, SmCE could be utilized as a vaccine candidate for schistosomiasis [36].

### *Schistosoma mansoni*/*japonicum* Cathepsin B (SmCB1; SjCB2)

Another enzyme employed by cercariae in evading host dermal immune responses is cathepsin B, which is a cysteine protease secreted by cercariae of *S. mansoni* (SmCB1) and *S. japonicum* (SjCB2). The enzyme is present in the acetabular glands and ducts of the cercariae [135]. SjCB2 has been shown to inhibit host immune responses by degrading dermal antibodies including IgA, IgM, and IgG [135]. SmCB1 elicits a Th2 immune response, stimulating CD4^+^ T cells and IL-4, and induces an IgE-specific antibody response [28]. Hamsters immunized with SmCB1 had 75% protection against *S*. *mansoni*, characterized by an increase in IL-5 and IL-13 and a decrease in IFN-γ, indicating a typical Th2 immune response [114]. Although the mechanism of immune modulation by SmCB1 or SjCB2 remains unclear, it offers promising aspects for future vaccine development for schistosomiasis and autoimmune diseases.

### Immune modulatory effects of *Schistosoma* adult worm antigen

The tegument of the adult worm is enriched with immune modulatory factors, and it can rapidly rejuvenate following damage, a phenomenon aided by somatic stem cells [21]. The adult worm spends up to decades in the host as a foreign body. Therefore, the adult worm incorporates various mechanisms to escape host immune surveillance. One of the mechanisms the adult worm uses is to employ self-made elements, such as cystatin and integrin, which can inactivate macrophages and alter cytokine release in T-cells [12]. Another molecule is schixator, produced by *S. japonicum*, which has been shown to have an anti-thrombotic effect and can lead to lower bleeding risk in mice [31], suggesting its potential use as novel drug therapy against thrombotic diseases. Below is a review of different adult worm antigens and their immune regulatory effects.

### *Schistosoma mansoni* Kunitz type serine protease inhibitor (SmKI-1)

SmKI-1 is found in both larva and adult *S. mansoni* and comprises a Kunitz-type serine protease inhibitor motif (KD) and a C-terminus domain. The KD domain has been suggested to inhibit trypsin, chymotrypsin, and neutrophil elastase (NE), inhibiting neutrophil influx and reducing inflammation [83, 96]. In an acetaminophen-induced liver injury model, SmKI-1 significantly reduced neutrophil recruitment and elastase activity in the liver, resulting in lesser liver histopathology [83]. SmKI-1 treatment also reduces joint destruction in the monosodium urate-induced gout arthritis model by reducing leukocyte infiltration and synovial membrane hyperplasia [83]. Similar anti-inflammatory effects were observed in carrageenan-induced pleural cavity inflammation models [83].

### *Schistosoma mansoni* protein 29 (Sm29)

*Schistosoma mansoni* protein 29 (Sm29), located in the tegument of adult worms, have been shown to induce a tolerogenic profile on DCs [71], as indicated by increased expression of HLA-DR, CD83, CD80, CD86, IL-10, and IL-10 receptors. Sm29 also stimulates the differentiation of naïve T-cells into Treg cells, which is opposite from the Th2 inducing effect of crude SEA [71]. High levels of Sm29-specific IgG1 and IgG2a antibodies with a polarized Th1 immune profile have been associated with resistance to *Schistosoma* infection in endemic areas, emphasizing the potential of Sm29 as a vaccine candidate [14].

Evidence has supported the ability of Sm29 to suppress inflammatory responses in different diseases [15, 69, 71]. Sm29 improved airway inflammation by decreasing ovalbumin-specific IgE levels and increasing the numbers of CD4^+^FoxP3^+^ T cells [15]. Sm29 has been demonstrated to suppress inflammatory responses in leishmaniasis by increasing the numbers of CD4^+^CD25 and CD4^+^CTLA-4^+^ T cells, leading to increased levels of IL-10 [71]. *In vitro*, Sm29 has been shown to downregulate the inflammatory response induced by human T cell lymphocytic virus type 1 (HTVL-1) by stimulating IL-10 and suppressing IFN-γ [69]. These findings support using Sm29 as an immunotherapeutic agent for treating inflammatory diseases.

### *Schistosoma mansoni* Cyclophilin A (SmCyp)

*Schistosoma mansoni* cyclophilin A (SmCyp) is a 17–19 kDa proteome secreted by adult *S. mansoni* worms [39]. SmCyp has been shown to modulate immune responses by altering the pro-inflammatory cytokines of LPS-activated DCs and also increasing the expression of Tregs *in vitro* [39]. It has been demonstrated that SmCyp immunization increased SmCyp-specific antibodies and reduced worm burden following *Schistosoma* infection [27]. In addition, a study has shown that SmCyp immunization does not activate IgE responses, whereas many vaccines that trigger IgE induction are possibly linked to hypersensitivity reactions [53]. Hence, it is inferred that SmCyp exhibits excellent safety as a vaccine candidate.

### *Schistosoma japonicum* tetraspanin orphan receptor (SjTOR)

*Schistosoma japonicum* tetraspanin orphan receptor (SjTOR) is present in cercariae, schistosomula, and adult worm [73]. SjTOR has been shown to bind to complement C2 and modulate complement-mediated hemolysis in a dose-dependent manner, thereby escaping from bloodstream coagulation [73]. This antigen also induces specific IgG1 and IgG2a antibodies, reducing worm burden in mice [73]. Although further research is required, SjTOR has attributes for vaccine development for schistosomiasis and therapeutics for thrombotic diseases.

### Immune modulatory effects of *Schistosoma* miracidia antigens

A favorable miracidia infection depends on snail immune response against schistosome [92]. Snail-schistosome compatibility is species-specific; for example, *S. japonicum* infects *Oncomelania* snails, *S. haematobium* infects *Bulinus* snails, and *S. mansoni* infects *Biomphalaria* snails. Most of the knowledge on gastropod immunity to schistosomes has been based on the *Biomphalaria glabrata* (*B. glabrata*)*-S. mansoni* model, which has been studied extensively [92]. Miracidia employs three strategies to aid host immune evasion: molecular mimicry, polymorphic mucins, and larva transformation products (LTPs). A mass spectrometry analysis of the LTPs discovered various factors involved in immune modulation, including but not limited to proteases, protease inhibitors, ion-binding proteins, antioxidative enzymes, and venom allergen-like proteins [125]. These LTPs alter plasma and hemocytic function, affecting hemocyte-attracting chemokines and the production of reactive oxygen species [132]. Below we will discuss how LTPs and other secretory products modulate immune responses.

### *Schistosoma mansoni* venom allergen-like 9 (SmVAL9)

*Schistosoma mansoni* venom allergen-like 9 (SmVAL9) belongs to a group of SmVAL molecules comprising 29 members [131]. SmVAL9 is essential for parasite development and host interactions. Although it is found in both miracidia and sporocysts, it is present in higher concentrations in miracidia than in developing sporocysts [131].

While matrix metalloproteinases (MMPs) are metacin-like proteases that regulate tissue hemostasis and immunity [70], mammalian RTV-1 (a homolog of SmVAL9) has been shown to upregulate MMP-2 activity in glioma cells and control the growth, survival, and invasion of glioma cells [101]. SmVAL9 stimulates MMPs and tissue inhibitors of metalloproteinases (TIMPs) in murine bone marrow-derived macrophages [131], suggesting a pivotal role played by SmVAL9 in tissue organization during miracidia and sporocyst migration and invasion [131].

### *Schistosoma mansoni* polymorphic mucins (SmPoMucs)

*Schistosoma mansoni* polymorphic mucins (SmPoMucs) are a group of highly polymorphic and glycosylated proteins produced by miracidia and sporocysts that aid parasite survival [99]. SmPoMucs have been found to be associated with different somatic immune-modifying molecules, such as fibrinogen-related proteins (FREPs) and *B. glabrata* thioester-containing protein (BgTEP), which are capable of neutralizing SmPoMucs [47]. However, their high polymorphism is a superpower in establishing infection and escaping host immune surveillance, such as forming the humoral immune complex of FREPS/BgTEP [47]. SmPoMuc interaction and immune modulation of FREPs are major determinants of the incompatibility/compatibility status in the *S. mansoni*-*B. glabrata* model [94]. However, whether these proteins process any immune modulatory effects in humans remains unclear.

## Conclusion

Different developmental stages of the schistosome exhibit different immune modulatory effects on the host. Current studies suggest the potential role of schistosome-derived products for schistosomiasis vaccine and drug development for autoimmune diseases. Presently, studies have focused on enhancing our understanding of using SEA to combat immune-related diseases; SEA comprises all soluble components of the schistosome eggs, from hundreds to thousands of proteins, of which only a few have been identified and characterized. Although the exact mechanism has yet to be clarified, using SEA and other schistosome-derived antigens may revolutionize treatment of autoimmune or other immune-related diseases. However, it is important to note that some schistosome species, although the association is not reasonably sufficient and conflicting (except *S. haematobium*, which has already been found to correlate positively with bladder cancer [79]), might have a possible association with other types of cancer, such as liver cancer [111] and intestinal cancer [10]. Therefore, using *Schistosoma* antigens as an immunotherapeutic drug may require a deeper understanding of its mechanism. Yet, as may have been expected, the immune-regulatory characteristics of *Schistosoma* antigens are desirable for pre-clinical and clinical trials.
